# The relationship between thoracic kyphosis and age, and normative values across age groups: a systematic review of healthy adults

**DOI:** 10.1186/s13018-021-02592-2

**Published:** 2021-07-09

**Authors:** Mattia Zappalá, Stephen Lightbourne, Nicola R. Heneghan

**Affiliations:** 1 Physiotherapy Department, St John & St Elizabeth Hospital, 60 Grove End Rd., St John’s Wood, London, UK; 2 Bermuda Hospitals Board, King Edward Memorial Hospital, 7 Point Finger Road, Paget, DV 04 Bermuda; 3 Centre of Precision Rehabilitation for Spinal Pain (CPR Spine), School of Sport, Exercise and Rehabilitation Sciences, College of Life and Environmental Sciences, University of Birmingham, Birmingham, UK

**Keywords:** Kyphosis, Hyperkyphosis, Ageing, Normative value, Correlation, Thoracic spine, Gender, Ethnic group, Reference values, Healthy adults

## Abstract

**Background:**

Thoracic kyphosis is reported to increase with ageing. However, this relationship has not been systematically investigated. Peoples’ kyphosis often exceeds 40°, but 40° is the widely accepted cut-off and threshold for normality. Consequently, patients may be misclassified. Accurate restoration of kyphosis is important to avoid complications following spinal surgery. Therefore, specific reference values are needed. The objective of the review is to explore the relationship between thoracic kyphosis and age, provide normative values of kyphosis for different age groups and investigate the influence of gender and ethnicity.

**Methods:**

Two reviewers independently conducted a literature search, including seven databases and the Spine Journal, from inception to April 2020. Quantitative observational studies on healthy adults (18 years of age or older) with no known pathologies, and measuring kyphosis with Cobb’s method, a flexicurve, or a kyphometer, were included. Study selection, data extraction, and study quality assessment (AQUA tool) were performed independently by two reviewers. The authors were contacted if clarifications were necessary. Correlation analysis and inferential statistics were performed (Microsoft Excel). The results are presented narratively. A modified GRADE was used for evidence quality assessment.

**Results:**

Thirty-four studies (24 moderate-quality, 10 high-quality) were included (n = 7633). A positive moderate correlation between kyphosis and age was found (Spearman 0.52, p < 0.05, T5-T12). Peoples’ kyphosis resulted greater than 40° in 65% of the cases, and it was significantly smaller in individuals younger than 40 years old (x < 40) than in those older than 60 years old (x > 60) 75% of the time (p < 0.05). No differences between genders were found, although a greater kyphosis angle was observed in North Americans and Europeans.

**Conclusion:**

Kyphosis increases with ageing, varying significantly between x < 40 and x > 60. Furthermore, kyphosis appears to be influenced by ethnicity, but not gender. Peoples’ thoracic sagittal curvature frequently exceeds 40°.

**Trial registration:**

The review protocol was devised following the PRISMA-P Guidelines, and it was registered on PROSPERO (CRD42020175058) before study commencement.

**Supplementary Information:**

The online version contains supplementary material available at 10.1186/s13018-021-02592-2.

## Background

Kyphosis, the convex curvature of the thoracic spine is considered ‘normal’ between 20 and 40° [1]. Where this exceeds 40°, the curvature is described as hyperkyphosis. This is associated with a higher risk of falling, developing pulmonary dysfunctions, and poor quality of life [2–4]. Hyperkyphosis is also associated with a higher risk of mortality for any cause [2–4]. A prospective longitudinal study, which followed 610 women for over 13 years, found that people with a greater thoracic kyphosis, who previously sustained a vertebral fracture, have a 1.5 times higher risk of death than those who have a smaller kyphotic curvature [5]. Consequently, it has been suggested that thoracic kyphosis is an important parameter to monitor, especially in the elderly population, to detect more frail people who may be at higher risk of unfavourable health [5].

The prevalence of hyperkyphosis increases with age; 20–40% of people older than 60 years of age and 55% of those older than 70 years have a kyphosis exceeding 40° [2–4]. Consequently, hyperkyphosis has been associated with ageing [4]. However, the relationship between kyphosis and age has not been systematically investigated. Individual studies show conflicting results [6, 7], and evidence supporting this association is derived from narrative reviews [2–4], rather than methodologically rigorous systematic reviews [8].

Despite evidence suggesting that peoples’ kyphosis often exceeds 40° [2–4], this value is widely used in clinical practice as the cut-off for normality [3, 4]. Consequently, clinicians may find many of their patients present with hyperkyphosis. Several authors have highlighted the need for a more accurate threshold for diagnosing hyperkyphosis [2–4], and a recent narrative review proposed to move the cut-off of normality to 50° [2]. The Scoliosis Research Society suggests using a range of 20–60° instead [9]. However, since people of different age groups have different degrees of kyphosis [2, 3], moving the cut-off of normality to a higher value, or expanding its range, may not reduce the risk of misdiagnosis. For these reasons, and due to the importance of the thoracic curvature when restoring patients’ sagittal alignment during spinal corrective surgery, to avoid post-operative complications such as proximal junctional kyphosis [10], having specific age-related reference values of kyphosis may be useful.

### Objective

This systematic review aims to investigate the sagittal curvature of the thoracic spine of adults with no health conditions which may affect their thoracic kyphosis and do the following:

1. Explore the relationship between kyphosis and age
2. Provide reference values of kyphosis for different age groups
3. Examine data for differences between genders or ethnic groups

## Methods

### Protocol and registration

The review’s protocol followed the Preferred Reporting Items for Systematic Reviews and Meta-Analyses guidelines for protocols (PRISMA-P) [11] and was registered on PROSPERO (CRD42020175058). The methods were informed by the Cochrane Handbook [12]. The manuscript adhered to the PRISMA [13] and the Synthesis Without Meta-analysis (SWiM) guidelines [14] for reporting.

### Eligibility criteria

The research question was informed by the Sample, Phenomenon of Interest, Design, Evaluation, Research type (SPIDER) tool [15], whose details are in Table 1.

**Table 1:** Eligibility criteria

**Sample**	Adults (18+ years old) without osteoporosis; vertebral fractures; pain; Scheuermann’s disease; scoliosis; history of spinal surgery or trauma; history of prolonged steroid use; rheumatological conditions; cardiac, lung or autoimmune diseases; cancer; metastasis; inflammatory or neurological disorders; pregnancy; or any genetic conditions affecting their bones, muscles or cartilage
**Phenomenon of interest**	Individuals’ thoracic sagittal alignment
**Design**	Any research design
**Evaluation**	Cobb’s method, a flexicurve or a Debrunner’s kyphometer
**Research type**	Quantitative, not performing interventions

### Information sources

Two reviewers (MZ/SL) independently searched for eligible articles on MEDLINE, EMBASE and PsycINFO through Ovid, and on AMED, The Index of Chiropractic Literature and CINAHL through EBESCO, from inception to April 2020. The Spine Journal, the reference list of the studies included in the review, and grey literature on SIGLE, through Open Grey, were also searched. The research was limited to studies published in English.

### Search

Keyword selection was informed by scoping review and researcher expertise (NRH). The search strategy was individualised for each database, combining keywords, Medical Subject Headings, and Boolean operators, and following consultation with a librarian. Keywords selected were middle back, dorsal spine, middle spine, mid-back, thoracic spine, kyphosis, hyperkyphosis, Dowager’s hump, hunchback, rounded back, and sagittal curvature (see Additional file 1 for search strategy examples).

### Study selection

The screening process was conducted independently by MZ and SL, then agreement was sought. In case of disagreement, a third reviewer (NRH) acted as a moderator. The studies were screened from their title and abstract first, then from their full text [8].

### Data collection

The data collection process was informed by the Cochrane Handbook [16]. The data extraction form was piloted with data extraction performed independently by MZ and SL and then cross-checked. If further information was necessary to reach a consensus among the research team, the authors were contacted by MZ.

### Data items

Data extraction was informed by the recommendations for reviews in clinical anatomy [8]. This included study title, author’s name, publication year, method for measuring kyphosis, degrees of kyphosis and range, sample size, age, age range, gender, body mass index, the standard deviation (SD) of the measures and ethnicity, defined as a group of people sharing cultural, geographical and social attributes.

### Risk of bias in individual studies

The studies’ quality assessment was performed independently by MZ and SL; NRH acted as a moderator in case of disagreement. The Anatomical Quality Assessment (AQUA) tool, devised for assessing the quality of anatomical studies [17], was used. As suggested by Chhapola et al. [18], a supplementary table to improve the tool’s performance was created (see Additional file 2). The AQUA tool is composed of 5 domains (i.e. objective(s) and subject characteristics, study design, methodology characterisation, descriptive anatomy, reporting of results); each of them has a specific set of questions whose answers could be either yes, no or unclear to enable the readers to evaluate the study’s quality. Currently, only indications about how to evaluate each individual domain of the AQUA tool exist. To be considered at low risk of bias in a single domain, the study must receive yes answers to all the questions of that specific domain; otherwise, the study would be considered at high risk [17]. Each domain was evaluated following the procedure just described. However, since no guidance exists on how to classify the overall quality of the evaluated study, the research team agreed that for a study to be considered, overall, high-quality, this must be at low risk of bias in all five domains. If at low risk in three or four domains they were considered moderate-quality, otherwise low-quality. The tool was then piloted before study commencement by MZ and SL on five articles and interrater agreement computed according to McHugh [19]. Perfect agreement was achieved (κ = 1).

### Summary measures

Data was analysed with Microsoft Excel of the Microsoft Office 365 package. Since kyphosis varies depending on the body references used to calculate it [6, 20], analysis was performed comparing the measurements for the same body references.

The mean kyphosis and age were used for correlation analysis. Either the Pearson’s or Spearman’s correlation coefficient was computed, depending on whether the data were normally distributed or not. Data distribution was investigated with the Kolmogorov-Smirnov test, and correlation was interpreted as recommended [21].

The means and their precision estimates were used to calculate the reference/normative values, or ranges, of kyphosis for each age group. Since SDs represent the dispersion of the values around their means, whereas confidence intervals are used to assess a treatment’s efficacy [22], SDs were deemed to be more appropriate to establish ranges. The mean kyphosis was utilised for group comparisons. Previous evidence regarding the relationship between kyphosis and age [2, 4, 6] was used to create the groups for analysis. These were people younger than 40 years old (x < 40), people between 40 and 60 (40 < x < 60), people older than 60 (x > 60), people younger than 50 (x < 50), and those older than 50 years old (x > 50). Inferential statistics was performed using the independent two-tailed t-test, for two group comparisons (x < 50, x > 50), or one-way ANOVA, for multiple group comparison (x < 40, 40 < x < 60, x > 60). Gender and ethnic group differences were investigated comparing each individual age group using the independent two-tailed t-test. Levene’s test was used to assess between groups’ equality variances. The selected alpha level was 0.05, and the Bonferroni correction was applied for post hoc analysis, after ANOVA, to reduce the chances of type I error [23, 24].

### Synthesis of results and risk of bias across studies

Since important clinical and methodological heterogeneity were observed during the scoping review, meta-analysis was not performed [25]. Data were synthesised narratively, and descriptive statistics presented [26]. The overall level of evidence was evaluated using a modified Grading of Recommendations, Assessment, Development and Evaluation (GRADE) system [27]. Whilst limited to observational studies, if the results were consistent (> 80% concordant results) [28], precise, and obtained predominantly from high-quality studies, the overall quality was upgraded from low to moderate. For correlation analysis, consistency was assessed by evaluating the direction of the correlation (positive or negative). For the reference values and for gender and ethnic group comparisons, statistical significance between groups’ means was used. Correlation analysis to be precise must be statistically significant, whereas for the normative values and for gender and ethnic group comparisons, the ranges of the groups with statistically significant different means must not overlap. Furthermore, their difference must be greater than the standard error of measurements for the modality employed to calculate kyphosis. These values were 2.4° for the kyphometer [29], 0.4 cm for the flexicurve [30], and 3° for Cobb’s method [7]. If the results were inconsistent, imprecise and coming primarily from low-quality studies, the results’ quality was downgraded to very low.

## Results

### Study selection

**Fig. 1:**
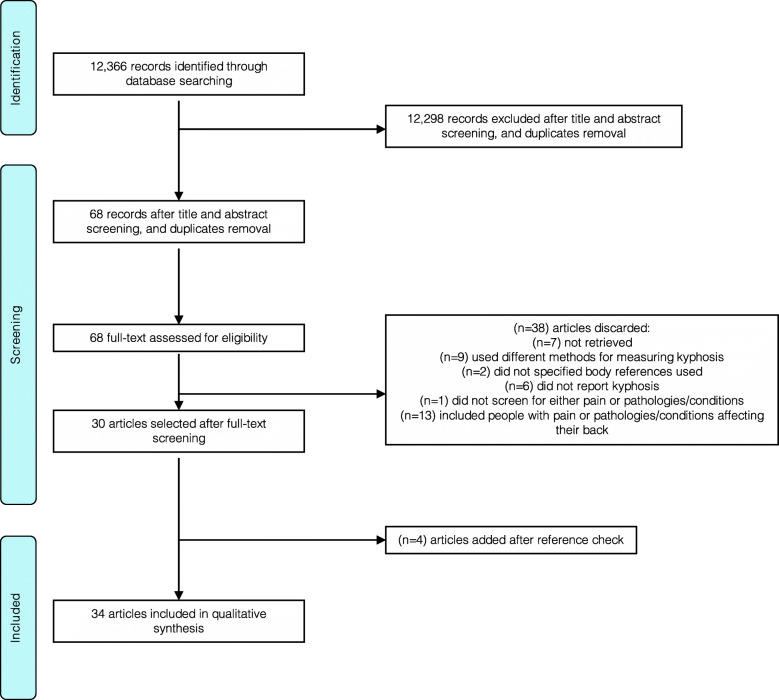
PRISMA flowchart

A total of 12,366 studies were retrieved, and 68 selected for full-text screening. Thirty-eight studies were excluded after the full-text screening, and four added following reference review, resulting in a total of 34 studies included in the review [6, 7, 20, 31–61] (Fig. 1).

### Study characteristics and individual studies results

Details about the included studies are in Table 2. From 7633 participants, the age range was 18–95 years old. Kyphosis was measured between C7–T12 (n = 220), T1–T12 (n = 2154), T2–T12 (n = 212), T3–T12 (n = 101), T4–T12 (n = 1617) and T5-T12 (n = 4018). Kyphosis was measured with a flexicurve in 293 individuals. Most studies used Cobb angle with just two (n = 293) studies using a flexicure [47, 52].

**Table 2:** Study characteristics and individual studies results

Authors and year	Type of measurement	Results	Sample size	Age (SD)	Age range	Gender	Ethnicity	BMI (SD)		Study design
		Kyphosis (SD) (degrees/cm)								
Amabile et al. 2016 [56]	Cobb T1–T12	49 (±13)	69	26.3	18–40	Mix	Europeans			Retrospective, observational
	Cobb T4–T12	35.1 (±11.5)								
Bakouny et al. 2017 [37]	Cobb T1–T12	51 (±7.9) 47.7 (±9.1) 49.4 (±8.6)	48 44 92	21.5 21.5 21.5	18–28 18–28 18–28	Male Female Mix	Asians	25.1 (±3.3) 21.8 (±2.4) 23.5 (±3.4)		Prospective cross-sectional cohort study, observational
	Cobb T2–T12	50.8 (±8.8) 47.4 (±10.1) 49.2 (±9.5)								
	Cobb T4–T12	43.1 (±8.8) 41 (±10.4) 42.1 (±9.6)								
Bassani et al. 2019 [20]	Cobb T1–T12	49.4 (±13.6) 54.4 (±13.2) 63.3 (±11.3)	44 83 27	66 74 83	60–69 70–79 x > 79	Mix	Europeans	25 (±3) 26 (±3) 25 (±3)		Prospective cross-sectional cohort study, observational
	Cobb T4–T12	40.8 (±10) 48 (±12.1) 57.7 (±10.8)								
Endo et al. 2014 [46]	Cobb T4–T12	27.5 (±9.6)	86	35.9 (±11.1)	23–59	Mix	Asians			Prospective cross-sectional cohort study, observational
Endo et al. 2016 [40]	Cobb T4–T12	30.5 (±8.3) 24.1 (±10.4) 27.8 (±9.7)	30 22 52	35.2 (±10.2) 35.8 (±13.6) 35.4 (±11.6)	22–50 22–50 22–50	Male Female Mix	Asians	21 (±2.7) 21 (±2.7) 21 (±2.7)		Prospective cross-sectional cohort study, observational
Gangnet et al. 2006 [54]	Cobb T1–T12	44.5 (±11.3)	34	30		Mix	Europeans			Retrospective, observational
	Cobb T4–T12	35.7 (±7.7)								
Gelb et al. 1995 [7]	Cobb T5–T12	36 (±11) 32 (±10) 36 (±11) 33 (±14)	27 27 37 9		40–49 50–59 60–69 x > 70	Mix	North Americans			Prospective cross-sectional cohort study, observational
Granito et al. 2014 [48]	Cobb T1–T12	29.99 (±5.12) 36.11 (±3.71) 37.14 (±2.67)	10 10 10	24.6 (±2.27) 43.5 (±2.88) 62.4 (±2.67)		Female	South Americans	20.9 (±1.45) 22.79 (±2.72) 26.2 (±2.32)		Prospective cross-sectional cohort study, observational
Hammerberg et al. 2003 [61]	Cobb T1–T12	52.5 (±12.2)	50	76.3	70–85	Mix	North Americans			Prospective cross-sectional cohort study, observational
Hasegawa et al 2016 [51]	Cobb T1–T12	41.5 (±9.9)	126	39.4 (±11.3)		Mix	Asians	21.1 (±2.4)		Prospective cross-sectional cohort study, observational
	Cobb T4–T12	29.6 (±9.2)								
Hasegawa et al. 2017 [53]	Cobb T1–T12	43.7 (±9) 41 (±10.2) 41.8 (±19.58)	40 96 136	40 39.6 39.7		Male Female Mix	Asians	21.4 21.4 21.4		Prospective cross-sectional cohort study, observational
Hinman 2004 [47]	Flexicurve	12.19 (±3.71) (cm) 10.02 (±2.43) (cm)	25 26	29.2 72.3	21–51 66–68	Female	North Americans			Prospective cross-sectional cohort study, observational
Hu et al. 2016 [42]	Cobb T5–T12	24.6 (±9.4) 23.5 (±8.5) 24.2 (±9)	161 111 272	23.2 (±3.4) 23.2 (±5.4) 23.2 (±4.4)	18–45 18–45 18–45	Male Female Mix	Asians			Prospective cross-sectional cohort study, observational
Hu et al. 2020 [32]	Cobb T5–T12	24.7 (±9.5) 23.8 (±8.5) 22.6 (±7.7) 23.9 (±8.5) 25.2 (±7.5) 27.2 (±8.5) 26.8 (±8.5) 27.4 (±8.5) 28.5 (±7.2) 27.5 (±8.5) 28.6 (±8.8) 28.6 (±8.5) 30.8 (±13.2) 29 (±8.5)	40 46 40 41 40 42 41 45 40 44 41 40 40 44		20–29 20–29 30–39 30–39 40–49 40–49 50–59 50–59 60–69 60–69 70–79 70–79 x > 80 x > 80	Male Female Male Female Male Female Male Female Male Female Male Female Male Female	Asians			Prospective cross-sectional cohort study, observational
Iyer et al. 2016 [6]	Cobb T2–T12	46.3 (±6.3) 39.6 (±14.7) 41.2 (±13.3) 37.3 (±10.7) 46 (±11) 43.2 (±11.4) 59.6 (±21) 40.4 (±6.8) 44.5 (±13.2) 48.5 (±15.4) 51.4 (±10.7) 50.9 (±11.2) 52.4 (±11.3) 44.7 (±14.5) 47.7 (±13.6) 46 (±15.7) 54.2 (±16.4) 49.8 (±16.1)	5 16 21 6 13 19 4 15 19 3 13 16 11 16 27 9 9 18		21–30 21–30 21–30 31–40 31–40 31–40 41–50 41–50 41–50 51–60 51–60 51–60 61–70 61–70 61–70 x > 71 x > 71 x > 71	Male Female Mix Male Female Mix Male Female Mix Male Female Mix Male Female Mix Male Female Mix	North Americans	26.5 (±6.6) 26.5 (±6.6) 26.5 (±6.6) 28.2 (±7) 28.2 (±7) 28.2 (±7) 31.4 (±7.8) 31.4 (±7.8) 31.4 (±7.8) 28.2 (±6.2) 28.2 (±6.2) 28.2 (±6.2) 27.4 (±2.7) 27.4 (±2.7) 27.4 (±2.7) 26.8 (±4) 26.8 (±4) 26.8 (±4)		Prospective cross-sectional cohort study, observational
Iyer et al. 2016 [6]	Cobb T5–T12	34.8 (±7.7) 27 (±11.4) 28.9 (±11) 27.8 (±9.9) 37.3 (±10.7) 31.2 (±9.3) 46 (±22.3) 29.1 (±6.4) 32.7 (±12.8) 39.9 (±11.2) 37.9 (±12.8) 38.3 (±12.2) 39.5 (±8.7) 34.4 (±15.6) 36.4 (±13.3) 33.3 (±13.3) 44 (±16) 38.3 (±15.2)	5 16 21 6 13 19 4 15 19 3 13 16 11 16 27 9 9 18		21–30 21–30 21–30 31–40 31–40 31–40 41–50 41–50 41–50 51–60 51–60 51–60 61–70 61–70 61–70 x > 71 x > 71 x > 71	Male Female Mix Male Female Mix Male Female Mix Male Female Mix Male Female Mix Male Female Mix	North Americans		26.5 (±6.6) 26.5 (±6.6) 26.5 (±6.6) 28.2 (±7) 28.2 (±7) 28.2 (±7) 31.4 (±7.8) 31.4 (±7.8) 31.4 (±7.8) 28.2 (±6.2) 28.2 (±6.2) 28.2 (±6.2) 27.4 (±2.7) 27.4 (±2.7) 27.4 (±2.7) 26.8 (±4) 26.8 (±4) 26.8 (±4)	Prospective cross-sectional cohort study, observational
Janssen et al. 2009 [36]	Cobb T4–T12	37 (±7.3) 35 (±10)	30 30	27 26		Male Female	Europeans		21.9 21.4	Prospective cross-sectional cohort study, observational
Kim et al. 2014 [31]	Cobb T5–T12	21.1 (±7.8) 30.1 (±8.6)	184 158	21.2 63.8	19–28 53–79	Male	Asians		22.4 (±2.1) 23.9 (±2.9)	Prospective cross-sectional cohort study, observational
Korovessis et al. 1998 [44]	Cobb T4–T12	41.8 (±13)	99	52.7 (±15)	20–79	Mix	Europeans			Prospective cross-sectional cohort study, observational
Lafage et al. 2019 [55]	Cobb T1–T12	49.5 (±13.3)	119	50.8 (±17)	22–78	Mix	North Americans		28 (±6)	Retrospective, observational
	Cobb T4–T12	41.5 (±12.7)								
	Cobb T5–T12	36.1 (±12.5)								
Lee et al. 2015 [49]	Cobb T4–T12	28.5 (±9)	77	31.5 (±7.6)	21–50	Mix	Asians		22.6 (±3.7)	Prospective cross-sectional cohort study, observational
Le Huec et al. 2016 [38]	Cobb T1–T12	41.6 (±9.9)	131	35	18–76	Mix	Asians			Prospective cross-sectional cohort study, observational
	Cobb T4–T12	29.9 (±9.4)								
	Cobb T1–T12	41.1 (±9.8)	147	39.6	18–76	Mix	Europeans			
	Cobb T4–T12	33.7 (±8.9)								
Oe et al. 2015 [59]	Cobb T5–T12	28.3 (±5.8) 30.3 (±13.1) 32.9 (±11.1) 33.4 (±13.4) 33.8 (±11.3) 36.7 (±14.5) 38.8 (±12.6) 40.4 (±18.7)	14 22 73 101 108 203 68 67	50 50 60 60 70 70 80 80	50 50 60 60 70 70 80 80	Male Female Male Female Male Female Male Female	Asians		25.2 (±2.8) 21.3 (±2.8) 23.1 (±3.3) 22.8 (±3.3) 22.7 (±2.6) 22.3 (±2.7) 22.1 (±2.8) 22.3 (±3.5)	Prospective cross-sectional cohort study, observational
Park et al. 2013 [58]	Cobb T1–T12	38.31 (±12.12) 38.69 (±11.08) 39 (±11.52) 41 (±8.05) 26.33 (±21.53) 33.37 (±17.94)	25 25 50 25 25 50	23.4 23.4 23.4 65.8 65.8 65.8	20–29 20–29 20–29 60–74 60–74 60–74		Male Female Mix Male Female Mix	Asians	20.8 (±3.2) 20.8 (±3.2) 20.8 (±3.2) 23.1 (±3.7) 23.1 (±3.7) 23.1 (±3.7)	Retrospective, observational
Pavlovic et al. 2013 [52]	Flexicurve	3.2 (±2.2) (cm) 3.4 (±2.2) (cm)	104 138	39.5 52 (±4.6)			Female	North Americans		Prospective cross-sectional cohort study, observational
Schwab et al. 2006 [50]	Cobb T4–T12	38 (±12) 37 (±9) 44 (±12)	25 24 22	29.8 (±5.8) 47.3 (±7.22) 70.8 (±5.2)	21–40 41–60 x > 60		Mix	North Americans		Prospective cross-sectional cohort study, observational
Sudhir et al. 2016 [39]	Cobb T3–T12	32.55 (±10.93) 33.91 (±11.26) 31.17 (±10.5) 30.24 (±9.6) 35 (±11.75)	101 51 50 52 49	44.91 (±15.81) 48.59 (±13.23) 47.16 (±17.41) 32.17 (±10.4) 58.43 (±6.6)	18–79 18–73 18–79 18–48 50–79		Mix Female Male Mix Mix	Asians		Prospective cross-sectional cohort study, observational
Uehara et al. 2019 [41]	Cobb T5–T12	25 (±8) 27 (±9) 29 (±8) 31 (±10) 31 (±10) 30 (±11) 31 (±13) 33 (±19)	50 47 53 61 55 54 45 48	50 50 60 60 70 70 80 80	50 50 60 60 70 70 80 80		Male Female Male Female Male Female Male Female	Asians	22.7 (±2.9) 22.2 (±2.8) 24.1 (±2.1) 22.3 (±2.8) 22.5 (±3.4) 22.6 (±3.2) 22.4 (±2.8) 23.1 (±3.3)	Prospective cross-sectional cohort study, observational
Urrutia et al. 2014 [60]	Cobb T5–T12	36	760	66.6 (±11)			Mix	South Americans		Prospective cross-sectional cohort study, observational
Vialle et al. 2005 [57]	Cobb T4–T12	40.6 (±10)	300	35.4 (±12)	20–70		Mix	Europeans	23.5 (±3)	Prospective cross-sectional cohort study, observational
Yang et al. 2017 [34]	Cobb T1–T12	36.33 (±10.25) 39.28 (±9.58) 35.78 (±8.9) 36.72 (±11.11)	311 29 128 183	46.2 40.3 40.01 50.52	18–78 22–74	Mix Mix Male Female	Asians		Retrospective, observational.	
Yeh et al. 2018 [35]	Cobb T5–T12	35 (±10) 32 (±13) 31 (±13)	114 135 143	28 (±7) 52 (±5) 69 (±6)	20–40 41–60 61–80	Mix	Asians		Prospective cross-sectional cohort study, observational	
Yokoyama et al. 2017 [33]	Cobb C7–T12	38.8 (±10.4)	220	59	20–95	Mix	Asians		Prospective cross-sectional cohort study, observational	
Yukawa et al. 2018 [43]	Cobb T1–T12	34.9 (±8.1) 33.9 (±9.1) 37.3 (±9.1) 33.4 (±9) 35.9 (±8.5) 35.9 (±10.4) 39.4 (±10.5) 35.9 (±10.7) 39.7 (±10) 36 (±11.7) 35 (±11.8) 34.8 (±10.5)	48 53 51 50 50 57 56 51 50 60 50 50	20 20 30 30 40 40 50 50 60 60 70 70	20 20 30 30 40 40 50 50 60 60 70 70	Male Female Male Female Male Female Male Female Male Female Male Female	Asians		Prospective cross-sectional cohort study, observational	
Zhu et al 2014 [45]	Cobb T5–T12	27.6 (±8.7) 28.1 (±10.6) 27.8 (±11.4)	104 156 260	33.8 (±11.6) 34.6 (±10.8) 34.3 (±12.6)		Male Female Mix	Asians		Cross-sectional cohort study, observational	

*SD* standard deviation

### Risk of bias within studies

Ten of the studies were high-quality [20, 31, 32, 36, 44–47, 57, 61], 24 were moderate-quality [6, 7, 33–35, 37–43, 48–56, 58–60] and none low-quality (see Table 3 for details). The most frequent limitation regarded studies’ methodology, with 12 studies [33, 34, 37–39, 42, 43, 48, 52, 53, 59] not reporting the accuracy of their measures. This limitation equally affected all measurement types.

**Table 3:** Risk of bias within studies

Authors and year		Q1	Q2	Q3		Q1	Q2	Q3	Q4		Q1	Q2	Q3	Q4	Q5		Q1	Q2	Q3	Q4		Q1	Q2	Q3	Q4		1	2	3	4	5		
Amabile et al. (2016) [56]	**Domain 1—objective(s) and subject characteristics**	Y	Y	Y	**Domain 2—study design**	Y	Y	Y	Y	**Domain 3—methodology characterisation**	Y	N	Y	Y	Y	**Domain 4—descriptive anatomy**	Y	Y	Y	Y	**Domain 5—reporting of results**	Y	Y	N	Y	**Domain results**	L	L	H	L	H	**Overall quality**	M
Bakouny et al. (2017) [37]		Y	Y	Y		Y	Y	Y	Y		Y	N	Y	N	Y		Y	Y	Y	Y		Y	Y	Y	Y		L	L	H	L	L		M
Bassani et al. (2019) [20]		Y	Y	Y		Y	Y	Y	Y		Y	Y	Y	Y	Y		Y	Y	Y	Y		Y	Y	Y	Y		L	L	L	L	L		H
Endo et al. (2014) [46]		Y	Y	Y		Y	Y	Y	Y		Y	Y	Y	Y	Y		Y	Y	Y	Y		Y	Y	Y	Y		L	L	L	L	L		H
Endo et al. (2016) [40]		Y	Y	Y		Y	Y	Y	Y		Y	N	Y	Y	Y		Y	Y	Y	Y		Y	Y	Y	Y		L	L	H	L	L		M
Gangnet et al. (2006) [54]		Y	Y	Y		Y	Y	Y	Y		Y	N	Y	Y	Y		Y	Y	Y	Y		Y	Y	N	Y		L	L	H	L	H		M
Gelb et al. (1995) [7]		Y	Y	Y		Y	Y	Y	Y		Y	Y	Y	Y	Y		N	Y	Y	Y		Y	Y	Y	Y		L	L	L	H	L		M
Granito et al. (2014) [48]		Y	Y	Y		Y	Y	Y	Y		Y	Y	Y	N	Y		Y	Y	Y	Y		Y	Y	N	Y		L	L	H	L	H		M
Hammerberg et al. (2003) [61]		Y	Y	Y		Y	Y	Y	Y		Y	Y	Y	Y	Y		Y	Y	Y	Y		Y	Y	Y	Y		L	L	L	L	L		H
Hasegawa et al. (2016) [51]		Y	Y	Y		Y	Y	Y	Y		Y	N	Y	N	Y		Y	Y	Y	Y		Y	Y	Y	Y		L	L	H	L	L		M
Hasegawa et al. (2017) [53]		Y	Y	Y		Y	Y	Y	Y		Y	N	Y	Y	Y		Y	Y	Y	Y		Y	Y	Y	Y		L	L	H	L	L		M
Hinman (2004) [47]		Y	Y	Y		Y	Y	Y	Y		Y	Y	Y	Y	Y		Y	Y	Y	Y		Y	Y	Y	Y		L	L	L	L	L		H
Hu et al. (2016) [42]		Y	Y	Y		Y	Y	Y	Y		Y	N	Y	N	Y		Y	Y	Y	Y		Y	Y	Y	Y		L	L	H	L	L		M
Hu et al. (2020) [32]		Y	Y	Y		Y	Y	Y	Y		Y	Y	Y	Y	Y		Y	Y	Y	Y		Y	Y	Y	Y		L	L	L	L	L		H
Iyer et al. (2016) [6]		Y	Y	Y		Y	Y	Y	Y		Y	Y	Y	Y	Y		Y	Y	N	N		Y	Y	N	Y		L	L	L	H	H		M
Janssen et al. (2009) [36]		Y	Y	Y		Y	Y	Y	Y		Y	Y	Y	Y	Y		Y	Y	Y	Y		Y	Y	Y	Y		L	L	L	L	L		H
Kim et al. (2014) [31]		Y	Y	Y		Y	Y	Y	Y		Y	Y	Y	Y	Y		Y	Y	Y	Y		Y	Y	Y	Y		L	L	L	L	L		H
Korovessis et al. (1998) [44]		Y	Y	Y		Y	Y	Y	Y		Y	Y	Y	Y	Y		Y	Y	Y	Y		Y	Y	Y	Y		L	L	L	L	L		H
Lafage et al. (2019) [55]		Y	Y	Y		Y	Y	Y	Y		Y	N	Y	N	Y		Y	Y	Y	Y		Y	Y	Y	Y		L	L	H	L	L		M
Lee et al. (2015) [49]		Y	Y	Y		Y	Y	Y	Y		Y	Y	Y	Y	Y		Y	Y	Y	Y		Y	Y	N	Y		L	L	L	L	H		M
Le Huec et al. (2016) [38]		Y	Y	Y		Y	Y	Y	Y		Y	N	Y	N	Y		Y	Y	Y	Y		Y	Y	Y	Y		L	L	H	L	L		M
Oe et al. (2015) [59]		Y	Y	Y		Y	Y	Y	Y		Y	Y	Y	N	Y		N	Y	N	Y		Y	Y	Y	Y		L	L	H	H	L		M
Park et al. (2013) [58]		Y	Y	Y		Y	Y	Y	Y		Y	Y	Y	Y	Y		Y	Y	Y	Y		Y	Y	N	Y		L	L	L	L	H		M
Pavlovic et al. (2013) [52]		Y	Y	Y		Y	Y	Y	Y		Y	N	Y	N	Y		Y	Y	Y	Y		Y	Y	Y	Y		L	L	H	L	L		M
Schwab et al. (2006) [50]		Y	Y	Y		Y	Y	Y	Y		Y	N	Y	Y	Y		Y	Y	Y	Y		Y	Y	Y	Y		L	L	H	L	L		M
Sudhir et al. (2016) [39]		Y	Y	Y		Y	Y	Y	Y		Y	Y	Y	N	Y		N	Y	Y	Y		Y	Y	Y	Y		L	L	H	H	L		M
Uehara et al. (2019) [41]		Y	Y	Y		Y	Y	Y	Y		Y	Y	Y	Y	Y		N	Y	Y	Y		Y	Y	Y	Y		L	L	L	H	L		M
Urrutia et al. (2014) [60]		Y	Y	Y		Y	Y	Y	Y		Y	Y	Y	Y	Y		N	Y	Y	Y		Y	Y	Y	Y		L	L	L	H	L		M
Vialle et al. (2005) [57]		Y	Y	Y		Y	Y	Y	Y		Y	Y	Y	Y	Y		Y	Y	Y	Y		Y	Y	Y	Y		L	L	L	L	L		H
Yang et al. (2017) [37]		Y	Y	Y		Y	Y	Y	Y		Y	Y	Y	N	Y		Y	Y	N	Y		Y	Y	Y	Y		L	L	H	H	L		M
Yeh et al. (2018) [35]		Y	Y	Y		Y	Y	Y	Y		Y	Y	Y	Y	Y		Y	Y	N	Y		Y	Y	Y	Y		L	L	L	H	L		M
Yokoyama et al. (2017) [33]		Y	Y	Y		Y	Y	Y	Y		Y	Y	Y	N	Y		Y	Y	Y	Y		Y	Y	Y	Y		L	L	H	L	L		M
Yukawa et al. (2018) [43]		Y	Y	Y		Y	Y	Y	Y		Y	N	Y	N	Y		Y	Y	Y	Y		Y	Y	N	Y		L	L	H	L	H		M
Zhu et al. (2014) [45]		Y	Y	Y		Y	Y	Y	Y		Y	Y	Y	Y	Y		Y	Y	Y	Y		Y	Y	Y	Y		L	L	L	L	L		H

*Q* question, *N* no, *Y* yes, *H* high, *M* moderate, *L* low

### Relationship between kyphosis and age

Only studies measuring kyphosis using Cobb’s method were included in the analysis because of the greater sample size, which provides greater statistical power [23], and those using a flexicurve included only women, limiting their generalisability. No analysis was performed for C7–T12 and T3–T12 because data came from single studies.

A positive correlation between kyphosis and age was found (see Table 4). The strength of the correlation was moderate for T5–T12 (Spearman 0.52) and low for T4–T12 (Spearman 0.45). The sample size for T5–T12 was more than double that for T4–T12 [25], giving more confidence in the findings for T5–T12.

**Table 4:** Correlation analysis, normative values and between-group difference

		Body reference	Correlation (p-value)	Sample size		Age group	Mean (SD) (degrees)	Range (degrees)	Group comparisons (p-value)
All ethnicity All genders		T1–T12	0.23 (0.21)	2154	997	x < 40	39.8 (±5.87)	33.93–45.67	x < 40 – x > 60^b^ (0.015) x < 40 – 40 < x < 60 (0.32) 40 < x < 60 – x > 60^b^ (0.004) X < 50 – x > 50 (0.17)
					683	40 < x < 60	38.54 (±4.68)	33.86–43.22	
					474	x > 60	43.56 (±10.5)	33.08–54.04	
					1454	x < 50	38.94 (±5.18)	33.76–44.12	
					700	x > 50	43.11 (±9.56)	33.55–52.67	
		T2–T12	0.64 (0.12)	212	132	x < 40	44.53 (±4.16)	40.37–48.7	x < 40 – x > 60 (0.49) x < 40 – 40 < x < 60 (0.49) 40 < x < 60 – x > 60 (0.49) X < 50 – x > 50 (0.07)
					35	40 < x < 60	47.7 (±4.53)	43.17–52.23	
					61	x > 60	48.75 (±1.48)	47.27–50.23	
					151	x < 50	44.53 (±3.4)	41.13–47.92	
					61	x > 50	49.47 (±1.63)	47.84–51.09	
		T4–T12	0.45^a^ (0.048)	1617	1199	x < 40	33.88 (±4.88)	29–38.77	x < 40 – x > 60^b^ (0.00052) x < 40 – 40 < x < 60 (0.055) 40 < x < 60 – x > 60 (0.16) X < 50 – x > 50^a^ (0.0003)
					242	40 < x < 60	40.1 (±2.69)	37.41–42.79	
					176	x > 60	47.63 (±7.33)	40.29–54.96	
					1223	x < 50	34.11 (±4.77)	29.34–38.87	
					394	x > 50	45.63 (±6.47)	39.17–52.1	
		T5–T12	0.52^a^ (0.0001)	4018	1037	x < 40	26.32 (±4.33)	21.99–30.65	x < 40 – x > 60^b^ (0.00011) x < 40 – 40 < x < 60 (0.036) 40 < x < 60 – x > 60 (0.086) X < 50 – x > 50^a^ (0.001)
					644	40 < x < 60	30.31 (±4.31)	26–34.61	
					2337	x > 60	32.59 (±3.63)	28.96–36.22	
					1165	x < 50	27.45 (±4.6)	22.75–32.15	
					2853	x > 50	31.94 (±3.91)	28.03–35.85	
Ethnicity	North America (N.A.)	T2–T12	0.87^a^ (0.02)	120	40	x < 40	42.2 (±1.41)	40.79–43.61	
					35	40 < x < 60	47.7 (±4.52)	43.17–52.23	
					45	x > 60	48.75 (±1.48)	47.27–50.23	T1–T12
					59	x < 50	42.97 (±1.66)	41.3–44.63	Asia - Europe
					61	x > 50	49.47 (±1.63)	47.84–51.09	x < 50^a^ (0.036)
		T4–T12	0.82 (0.18)	190	49	x < 50	37.5 (±0.7)	36.79–38.21	x > 50^a^ (0.043)
					141	x > 50	42.75 (±1.77)	40.98–44.52	
		T5–T12	0.65^a^ (0.03)	339	40	x < 40	30.05 (±1.63)	28.42–31.68	
					208	40 < x < 60	35.02 (±2.62)	32.4–37.64	T4–T12
					91	x > 60	35.93 (±2.19)	33.73–38.12	N.A. - Europe
					86	x < 50	32.2 (±2.98)	29.22–35.18	x < 50 (0.5)
					253	x > 50	35.73 (±2.42)	33.31–38.15	x > 50 (0.5)
	Asia	T1–T12	− 0.18 (0.46)	1551	737	x < 40	39.2 (±5.07)	34.13–44.27	
					554	40 < x < 60	37.12 (±1.73)	35.39–38.85	
					260	x > 60	35.77 (±2.39)	33.39–38.16	
					1184	x < 50	38.48 (±4.37)	34.11–42.85	T5–T12
					367	x > 50	36.31 (±2.38)	33.93–38.69	N.A. - Asia
		T5–T12	0.72^a^ (0.0000006)	2919	997	x < 40	25.39 (±4.33)	21.06–29.71	x < 40 (0.19)
					436	40 < x < 60	27.69 (±2.26)	25.43–29.95	40 < x < 60^a^ (0.00013)
					1486	x > 60	31.76 (±3.49)	28.27–35.24	x > 60^a^ (0.033)
					1079	x < 50	25.55 (±3.86)	21.69–29.41	x < 50^a^ (0.0097)
					1486	x > 50	30.81 (±3.58)	27.23–34.39	x > 50^a^ (0.0017)
	Europe	T1–T12	0.8 (0.57)	404	250	x < 50	44.87 (±3.96)	40.9–48.83	
					154	x > 50	55.7 (±7.04)	48.66–62.74	
		T4–T12	0.88^a^ (0.00076)	863	610	x < 50	36.18 (±2.41)	33.77–38.6	
					253	x > 50	47.08 (±7.77)	39.31–54.84	
Gender	Female	T1–T12	− 0.17 (0.55)	724	278	x < 40	37.45 (±6.39)	31.06–43.83	
					301	40 < x < 60	36.16 (±0.39)	35.77–36.55	
					145	x > 60	33.57 (±4.92)	28.65–38.49	T1–T12
					345	x < 50	37.09 (±5.44)	31.65–42.52	Female-male
					379	x > 50	34.48 (±4.07)	30.41–38.55	x < 40 (0.52)
		T2–T12	0.6^a^ (0.15)	126	73	x < 40	44.33 (±4.16)	40.17–48.49	40 < x < 60 (0.27)
					28	40 < x < 60	45.9 (±7.78)	38.12–53.68	x > 60 (0.19)
					25	x > 60	49.45 (±6.76)	42.73–56.17	x < 50 (0.41)
					88	x < 50	43.35 (±3.92)	39.43–47.27	x > 50 (0.1)
					38	x > 50	50.1 (±4.88)	45.22–54.98	
		T5–T12	0.6^a^ (0.0024)	1254	383	x < 40	27.27 (±5.27)	21.99–32.54	
					184	40 < x < 60	29.82 (±4.16)	25.65–33.98	T2–T12
					687	x > 60	33.45 (±5.18)	28.27–38.63	Female-male
					440	x < 50	27.49 (±4.5)	22.98–31.99	x < 40 (0.92)
					814	x > 50	32.71 (±5.12)	27.58–37.83	40 < x < 60 (0.41)
	Male	T1–T12	0.03 (0.94)	571	172	x < 40	40.38 (±7.22)	33.15–47.6	x > 60 (0.97)
					274	40 < x < 60	38.7 (±3.74)	34.96–42.43	x < 50 (0.35)
					125	x > 60	38.57 (±3.16)	35.41–41.72	x > 50 (0.75)
					390	x < 50	39.56 (±5.83)	33.72–45.39	
					181	x > 50	38.78 (±2.61)	37.47–41.39	
		T2–T12	0.07 (0.88)	86	59	x < 40	44.8 (±6.87)	37.93–51.67	T5–T12
					7	40 < x < 60	54.05 (±7.85)	46.2–61.9	Female-male
					20	x > 60	49.2 (±4.53)	44.67–53.73	x < 40 (0.69)
					63	x < 50	48.5 (±9.29)	39.21–57.79	40 < x < 60 (0.62)
					23	x > 50	48.97 (±3.23)	45.74–52.19	x > 60 (0.4)
		T5–T12	0.51^a^ (0.0085)	1393	540	x < 40	26.17 (±4.9)	21.28–31.07	x < 50 (0.81)
					152	40 < x < 60	31.87 (±8.88)	22.99–40.75	x > 50 (0.57)
					701	x > 60	32.28 (±3.66)	28.62–35.99	
					584	x < 50	28.27 (±7.7)	20.55–35.99	
					809	x > 50	31.71 (±4.46)	27.24–36.17	

*x < 40* people younger than 40 years old, *40 < x < 60* people between 40 and 60 years old, *x > 60* people older than 60 years old, *x < 50* people younger than 50 years old, *x > 50* people older than 50 years old, *T* thoracic vertebra

^a^Statistical significance for p < 0.05 (t-test)

^b^Statistical significance for p < 0.0167 (ANOVA with Bonferroni post hoc correction)

### Normative values

Table 4 provides details of the mean kyphosis and normative values of kyphosis for different age groups, as well as between-group mean difference in kyphosis and the sample sizes. The same studies utilised to investigate the relationship between kyphosis and age were also used for calculating the reference values. Only 12 studies divided their sample by age groups [6, 7, 20, 31, 32, 35, 41, 43, 48, 50, 58, 59]. The ranges surpassed 40° in people < 60 years old 58.3% of the time and 75% in those older, questioning the accuracy of the current cut-off for normality.

### Gender and ethnic group differences

**Fig. 2:**
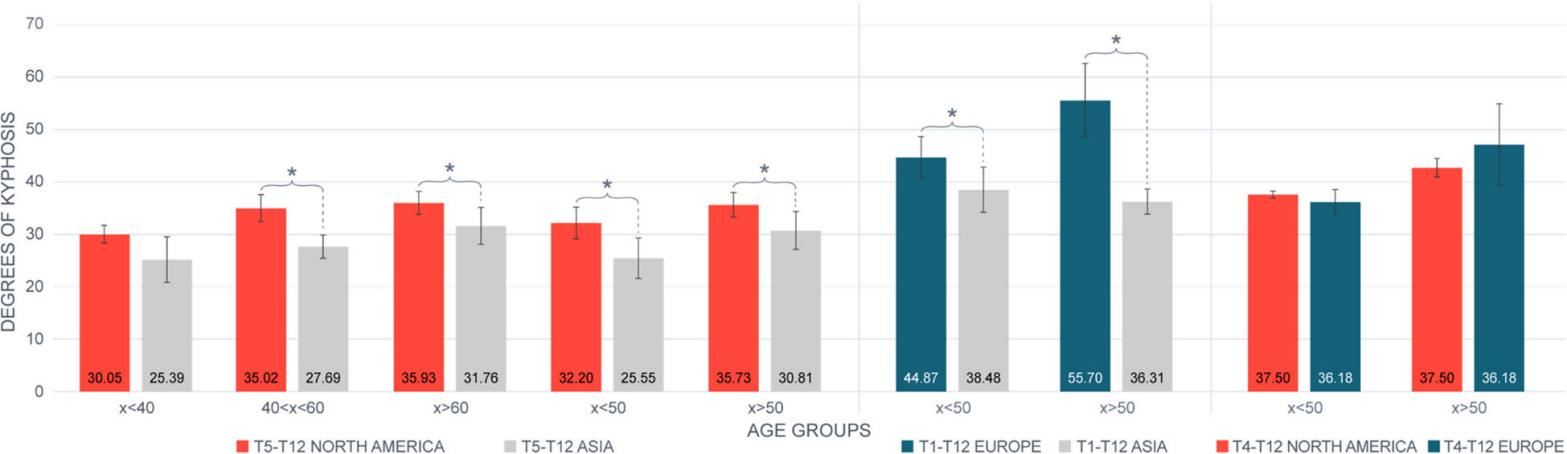
Ethnic group comparison. Data presented as mean standard deviation. *Statistical significance for p < 0.05 (t-test). x < 40, people younger than 40 years old; 40 < x < 60, people between 40 and 60 years old; x > 60, people older than 60 years old; x < 50, people younger than 50 years old; x > 50, people older than 50 years old

Fourteen studies specified sample ethnicity [20, 32, 34–38, 41–46, 59]; consequently, geographical provenience was the main determinant for ethnic group subdivision. Two studies were excluded from the sub-analysis between ethnicities. One study [60] did not divide their sample by age groups and did not report mean’s SD, whereas in the other study [48], the sample size was too small to exclude the chance of committing type II error. Fifteen of the included studies presented their results according to gender [6, 31, 32, 34, 36, 37, 40–43, 45, 48, 53, 58, 59], and only eight of those divided their sample by age [6, 31, 32, 41, 43, 48, 58, 59]. The results are reported in Table 4. No differences between genders were observed, but North Americans and Europeans showed a greater thoracic curvature than Asians (Fig. 2).

### Synthesis of results

There is moderate-quality evidence that a moderate positive correlation between age and kyphosis exists and that kyphosis does not differ between genders. The quality of the evidence for the normative values presented, and for the differences in kyphosis observed between ethnicities is low (Table 5).

**Table 5:** Synthesis of results

	Sample size	Number of studies	Results	Publication bias	Inconsistency (-)	Imprecision (-)	Indirectness	Study limitations/quality	Effect size/dose response	Possible confounding/bias	Overall quality
					Consistency (+)	Precision (+)					
**Correlation** **Kyphosis and age**	6793	30	Table 4	ND	(+) All correlations had positive sign	T1–T12: p > 0.05 (−) T2–T12: p > 0.05 (−) T4–T12: p < 0.05 (+) T5–T12: p < 0.05 (+)	None	(+) 9 HQ, 21 MQ	N/A		(+)(+)(+) Moderate
**Reference values**	6793	30	Table 4	ND	(−) Statistical significance was achieved in 6 out of 16 cases.	(−) Ranges did not overlap in 2 out of 6 cases. The between-range difference was always smaller than the SEM.	None	(+) 9 HQ, 21 MQ	N/A	(+) 12 articles divided sample by age groups	(+)(+) Low
**Ethnic group differences**	5788	25	Table 4	ND	(−) Statistical significance was achieved in 6 out of 9 cases.	(−) Ranges did not overlap in 2 out of 6 cases. In 1 of the 2 cases, the between-range difference was greater than the SEM.	None	(+) 8 HQ, 17 MQ	N/A	(+) 14 articles specified the ethnicity of their sample	(+)(+) Low
**Gender differences**	3937	13	Table 4	ND	(+) No between-group comparisons achieved statistical significance	(−)	None	(+) 3 HQ, 10 MQ	N/A		(+)(+)(+) Moderate

*ND* not detected, *HQ* high-quality, *MQ* moderate quality, *N/A* not applicable, *T* thoracic vertebra, *SEM* standard error of measurement

## Discussion

This is the first review exploring the relationship between kyphosis and age, in addition to providing normative kyphosis values for different ages, ethnic groups and genders. Findings evidence a positive correlation between kyphosis and age, as well as the influence of ethnicity on kyphosis. Gender, instead, does not appear to influence thoracic sagittal curvature.

### Relationship between kyphosis and age

Muscle strength, vertebral body shape and intervertebral disc morphology can affect kyphosis angle [3]. However, vertebral body shape and intervertebral disc morphology account for 86–93% thoracic spine curvature [62]. Disc morphology has a stronger negative correlation with ageing than vertebral morphology [62, 63]. Therefore, the increase in thoracic kyphosis observed with ageing may be related to the changes occurring in intervertebral discs. Most of these changes occur in the middle section of the thoracic spine [64], which can explain why statistical significance was reached only when kyphosis was measured from T4/5. For these reasons, and due to the technical difficulties with visualising the vertebrae above T4 from lateral radiographs [2], measuring kyphosis from T5 may provide more accurate measurements.

### Normative values

The normative values surpassed 40° in 65% of the analysis. This finding challenges the accuracy of the current threshold used for defining normality (i.e. 40°). This cut-off was first introduced by Roaf in 1960 [1], but without supporting evidence for it. Despite subsequent studies showing that healthy children, adolescents and adults could have thoracic curvatures exceeding 40° [6, 65], this value is still used in practice [3, 4]. Some authors suggested moving this cut-off to 50° [2]. However, even this suggestion may not decrease the chances of misclassifying patients, since 35% of the ranges presented in this review surpassed 50°. Using a range of 20–60° [9] may seem more appropriate, since the ranges provided never exceeded 60°. Nonetheless, people x < 40 appeared to have a significantly smaller kyphosis than those x > 60. Consequently, using the same reference values for both groups may lead to misclassification anyway. When kyphosis was measured between T4/5 and T12, its value significantly differed also between people x < 50 and x > 50. This may indicate a higher measurement precision when those body references were used. Thoracic kyphosis varied depending on the body references selected to calculate it, with a trend showing that including higher vertebrae leads to greater values. Therefore, using specific reference values, like those presented in this review, which account for age and body references, could be the most accurate alternative for clinicians.

### Gender and ethnic group differences

Thoracic kyphosis does not seem to be influenced by gender, since the between-group mean difference never reached statistical significance. Although the precision of the results could have been affected by the small number of studies subdividing their sample by age groups and gender, these findings align with previous evidence [7, 57].

Significant differences in kyphosis between the ethnic groups were seen, with Europeans and North Americans showing a greater kyphosis than Asians. Genetic differences may explain this result. A twins study found that thoracic kyphosis is influenced by genetics and that it also negatively correlates with bone mineral density [66], also related to genetics [67]. However, other lifestyle factors, such as sports, could also influence thoracic curvature [68], but no data were available to investigate those relationships. Since only 14 studies specified the sample ethnicity [20, 32, 34–38, 41–46, 59], people were grouped according to geography. This can represent a limitation since some areas have habitants from different socio-cultural backgrounds. Most of the studies that specified sample ethnicity included people from Asia [32, 34, 35, 37, 38, 41–43, 45, 59] or Europe [20, 36, 38, 44], which further affects the reliability of the results for North America.

### Strengths and limitations

This reviewed employed rigorous methods, with transparent reporting (PRISMA and SWiM guidelines), and a completed PRISMA checklist relative to this article can be found in Additional file 3. The main strength of this review lies in the high quality of studies included and the large sample size utilised for computing the values presented. These factors strengthen the confidence in study findings. No information about kyphosis measured with a kyphometer or flexicurve was provided because of poor information retrieval, perhaps due to the limited sensitivity of the search tool [15]. The AQUA tool was utilised to assess study quality, but data regarding its validity and reliability is lacking [17]. Since clinical and methodological heterogeneity can preclude a meta-analysis [25], and concerns regarding the reliability of the results of the meta-analysis carried out on observational studies exist [69], the authors considered a narrative synthesis most appropriate. Finally, the sample utilised to create the normative values presented was not randomly selected from the general population, but it was created by combining the samples of the individual studies included in the review, and this could represent a form of selection bias. However, the rigorous methodology employed, the size and the heterogeneity of the sample may partially mitigate this limitation.

### Clinical implications

Surgical interventions aiming to correct adult spinal deformities are recommended in those cases with progressive deformities, significant neural compromising, pain or functional limitations, and that did not respond to conservative management [9]. To help these patients, different surgical approaches are available, from minimally invasive operations, such as laminectomies, to deformity correction and vertebral fusion surgeries. These more invasive interventions may target only a limited and specific number of vertebrae in mild and moderate cases or extensive portions of the thoracic and lumbar spine in more severe cases [70], reaching as high as T3–T4 in some instances [71]. These more invasive interventions are associated with high risk of complications and worse functional outcomes if the surgical correction is suboptimal; thus, careful surgical planning is paramount [70]. Among the individual patient’s characteristics to be considered when planning for surgery, there are patient’s age [72] and ethnicity [71]; consequently, we believe that the normative values provided in this review, which account specifically for these characteristics, despite being supported by low-quality evidence, may prove beneficial in a clinical context. This information may help clinicians deciding and planning their interventions.

## Conclusion

This review provides evidence that a positive correlation between kyphosis and age exists. It also shows that thoracic kyphosis seems to not be influenced by gender, but to vary depending on ethnicity, age, and the body references used to measure it. The normative values of kyphosis currently used in clinical practice may not reduce the chances of misclassifying patients, since they do not account for those characteristics, and they may not be precise enough to correctly inform clinicians when planning and performing corrective spinal surgeries. Therefore, using specific reference values, such as those presented in this study, which account for body reference, age, and ethnicity, when assessing and treating patients may represent the most accurate solution for clinicians.

## Supplementary Information

**Additional file 1: Table S1**. Examples of search strategy.

**Additional file 2: Table S2**. Supplementary table for the AQUA tool.

**Additional file 3:.** PRISMA 2020 Checklist.

## Data Availability

The datasets used and/or analysed during the current study are available from the corresponding author on reasonable request.

## Abbreviations

ANOVA: Analysis of variance
AQUA: Anatomical Quality Assessment
C: Cervical vertebra
GRADE: Grading of Recommendations, Assessment, Development and Evaluation
H: High
HQ: High-quality
L: Low
M: Moderate
MQ: Moderate quality
N: No
N/A: Not applicable
ND: Not detected
PRISMA: Preferred Reporting Items for Systematic Reviews and Meta-Analyses
PRISMA-P: Preferred Reporting Items for Systematic Reviews and Meta-Analyses guidelines for protocols
Q: Question
SD: Standard deviation
SEM: Standard error of measurement
SPIDER: Sample, Phenomenon of Interest, Design, Evaluation, Research type
SWiM: Synthesis Without Meta-analysis
T: Thoracic vertebra
x < 40: People younger than 40 years old
x > 60: People older than 60 years old
x < 50: People younger than 50 years old
x > 50: People older than 50 years old
Y: Yes
40 < x < 60: People between 40 and 60 years old
