# Supplementary material for: The relationship between thoracic kyphosis and age, and normative values across age groups: a systematic review of healthy adults
Source: J Orthop Surg Res. 2021 Jul 9;16:447. doi: 10.1186/s13018-021-02592-2 (PMC8268398; doi:10.1186/s13018-021-02592-2)
Supplement: Supplementary file 1 — Additional file 1: Table S1. Examples of search strategy. [file 13018_2021_2592_MOESM1_ESM.docx]

**Additional file 1.** Examples of search strategy

| Search | **Keywords** | **hits** |
| --- | --- | --- |
| 1 | (MH "Spine+") OR (MH "Back") OR "middle back OR dorsal spine OR middle spine OR mid-back OR thoracic spine" | 42,691 |
| 2 | (MH "Spinal Curvatures+") OR (MH "Back") OR (MH "Kyphosis") OR "kyphosis OR hyperkyphosis OR dowager's hump OR hunchback OR rounded back OR sagittal curvature" | 12,111 |
| 3 | S1 AND S2 | 7,019 |
| 4 | S1 AND S2 limited to English language | 6,933 |
| 5 | S1 AND S2 limited to all adults | 3,315 |

CINAHL Plus (EBESCO) Advanced Search 15/03/2020

| Search | **keywords** | **hits** |
| --- | --- | --- |
| 1 | Middle back | 744 |
| 2 | Dorsal spine | 1687 |
| 3 | Middle spine | 60 |
| 4 | Mid-back | 1339 |
| 5 | Thoracic spine | 32006 |
| 6 | 1 OR 2 OR 3 OR 4 OR 5 | 35500 |
| 7 | Kyphosis | 40330 |
| 8 | Hyperkyphosis | 1530 |
| 9 | Dowager’s hump | 92 |
| 10 | Hunchback | 1412 |
| 11 | Rounded back | 217 |
| 12 | Sagittal curvature | 481 |
| 13 | 7 OR 8 OR 9 OR 10 OR 11 OR 12 | 42483 |
| 14 | 6 AND 13 | 5203 |
| 15 | Limit 14 to English | 5019 |
| 16 | Limit 15 to Humans | 4847 |
| 17 | Limit 16 to All Adults (19+ years) | 4710 |

MEDLINE + EMBASE + PsycINFO (Ovid) Advanced Search 25/02/2020

| Search | **Keywords** | **hits** |
| --- | --- | --- |
| 1 | (MH "Spine+") OR (MH "Back") OR "middle back OR dorsal spine OR middle spine OR mid-back OR thoracic spine" | 462 |
| 2 | (MH "Spinal Curvatures+") OR (MH "Back") OR (MH "Kyphosis") OR "Dowager's hump OR hunchbak OR rounded back OR sagittal curvature OR kyphosis OR hyperkyphosis" | 253 |
| 3 | S1 AND S2 | 38 |

AMED - The Allied and Complementary Medicine Database (EBESCO) Advanced Search 04/03/2020
