# Supplementary material for: The relationship between thoracic kyphosis and age, and normative values across age groups: a systematic review of healthy adults
Source: J Orthop Surg Res. 2021 Jul 9;16:447. doi: 10.1186/s13018-021-02592-2 (PMC8268398; doi:10.1186/s13018-021-02592-2)
Supplement: Supplementary file 2 — Additional file 2: Table S2. Supplementary table for the AQUA tool. [file 13018_2021_2592_MOESM2_ESM.docx]

**Additional file 2.** Supplementary table for the AQUA tool

| DOMAIN 1 – Objective(s) and subject characteristics | |
| --- | --- |
| YES (Low risk of bias) | NO (High risk of bias) |
| Aim of the study has to be clearly stated within the text. | The aim of the study is not clearly stated within the text |
| The process for selection and inclusion of participants into the study has to be described within the text or with graphs/flow charts. Participants have to be healthy and asymptomatic | Not clearly defining inclusion and exclusion criteria  Selection of convenient sample  No a-priori calculation  Inclusion of participants with spinal pain and/or affected by any of the conditions listed in the exclusion criteria of this study  Pre-screening of participants |
| Baseline demographic has to be clearly reported. | Baseline demographic is not clearly reported |

| DOMAIN 2 – Study design | |
| --- | --- |
| YES (Low risk of bias) | NO (High risk of bias) |
| The study has to be either observational cross-sectional or prospective longitudinal or retrospective observational to meet the criteria. | Any interventional studies will not meet the criteria. |
| accepted measures will be radiographic assessment, kyphotic index using a flexicurve and kyphometer. | Any other types of measurements, such as observation or occiput-to-wall distance will not be accepted. |
| To meet this criterion, the methods employed to collect the data have to be clearly described within the text in order to be reproducible | Methods to collect data are not clearly described, therefore they are not reproducible |
| Collection of data in static standing or supine lying using the methodology described in Q2 will be accepted. If novel methodologies are employed, they have to be clearly described in the text and they do not have to infer the degree of thoracic kyphosis by measuring other body areas (e.g. head or pelvis position, neck lordosis or low back lordosis). | Collection of data with subjects in positions other than static standing or supine lying won’t satisfy the criteria. Materials different from those ones described in Q2 won’t be accepted if not clearly described and if they give a degree of kyphotic curvature by assessing other body areas other than the thoracic spine. |

| DOMAIN 3 – Methodology characterisation | |
| --- | --- |
| YES (Low risk of bias) | NO (High risk of bias) |
| patient positioning, body reference used, mathematical analysis and methods to compute the data have to be clearly reported in the text. | patient positioning, body reference used, mathematical analysis and methods to compute the data are not clearly reported or there are inconsistencies in the methods used to collect and/or analyse data. |
| The characteristics of the researchers collecting the data have to be specified in the text | No references to the characteristics of the professional collecting the data |
| The characteristics of the machineries and/or instruments used to collect and computing the data have to be described in the text | The characteristics of the machines and/or instruments employed to collect the data are not specified in the text |
| Researchers collecting the data have to be familiar with the methodologies employed. Intra and/or inter-class correlation values, or standard measures of errors, have to be reported within the text. Body references selected have to be specified clearly in the text/images | No references to intra and/or inter-class correlation values are reported and when the body reference used for taking the measurements are not specified within the text/images |
| Images and/or description of the body references and methodologies employed to collect data and calculate results have to be presented in the text | No images and/or description of body references and methodologies employed to collect the data and compute the results are stated in the text |

| DOMAIN 4 – Descriptive anatomy | |
| --- | --- |
| YES (Low risk of bias) | NO (High risk of bias) |
| The authors have to clearly state that they included in their study only individuals with not known pathologies/conditions and no physical complaints. | Inclusion of individuals with known pathologies/conditions and/or suffering from pain. |
| Measurements of the thoracic spine curvature, in its entirety, or at least from T5 to T12, will be accepted if taken with either flexicurve, kyphometer or radiographic imager. The methods used have to be specified.  For sub-analysis a data on vertebral body height, intervertebral disc morphology and trunk muscle strength or morphology will be accepted. However, a clear description of how they were measured will be needed. | Measurements of only part of the thoracic spine (i.e. only thoraco-lumbar or cervico-thoracic junctions), or using methods other than radiographic images, flexicurve or kyphometer won’t be accepted unless specified in detail and assessing directly the thoracic spine. A lack of description of how measures of vertebral body height, intervertebral disc morphology and trunk muscle strength or morphology will not meet these criteria. |
| images reporting the procedures used to collect the degree of curvature of the thoracic spine have to be clear and understandable with a supporting detailed description which makes it comprehensible and reproducible | The images were not clear or supported by a detail description which make their reproduction impossible. |
| Anatomical variations and/or clear reasons for not taking the planned measurements have to be reported. If some data are excluded a clear explanation of the reasons behind that decision have to be provided. | Anatomical variations are not reported in enough details, and classify separately, when encountered. If data were excluded no details regarding that decision were provided. |

| DOMAIN 5 – Reporting of results | |
| --- | --- |
| YES (Low risk of bias) | NO (High risk of bias) |
| Appropriate statistical analysis are measures of central tendency, such as mean value, linear regression analysis and/or analysis of differences between and within groups. Their precision estimates have to be presented too. The type of statistical analysis employed has to be reported in the text/graph/descriptions. | The statistical methods used, and/or the statistics were not reported, or they were unclear |
| the data intended to be investigated have to be clearly stated and their values have to be reported within the text or in supporting tables/graphs/figures. | Results are not reported within the text or in tables/graphs/figures. |
| the number of results have to correspond to the subjects investigated. If they differ, the reasons for exclusion have to be specified within the text | The number of the results do not correspond with the number of subjects and no clear explanation as to why are provided |
| potential confounders, such as gender, have to be highlighted in the text or tables/graphs/figures. | Potential confounders are not reported either in the text or in tables/graphs/figures |
